# MEDUSA: An open-source and webcam based multispectral imaging system

**DOI:** 10.1016/j.ohx.2022.e00282

**Published:** 2022-02-22

**Authors:** Daniel Pineda, Juan Pérez, Daniel Gaviria, Karen Ospino-Villalba, Omar Camargo

**Affiliations:** a Facultad de Ciencias Universidad Nacional de Colombia Sede Medellín, Facultad de Ciencias Agrarias, Carrera 65 #59a-110, Medellín, Antioquia, Colombia; b Facultad de Ciencias Agrarias. Carrera 65 #59a-110, Medellín, Antioquia, Colombia

**Keywords:** Multispectral imaging, Python, Arduino

## Abstract

Multispectral imaging is at the forefront of contactless surface analysis. Standard multispectral imaging systems use sophisticated software, cameras and light filtering optics. This paper discloses the building of a customizable and cost-effective multispectral imaging and analysis system. It integrates a web camera, light emitting diodes (LEDs) lighting, a semisphere for even lightening, an open-source Arduino™ development board and a free Python application to automatically obtain and visually analyze multispectral images.

The device is hereafter called MEDUSA and its optical performance was tested for repeated Imaging consistency, visible and near infrared band sensitivity and lighting evenness. Four proof of concept tests were run in order to understand the advantageous use of this system, as compared to a simple visual score of diverse samples. Each of three qualitative tests used sets of 12 LED band spectral images to analyze ink changes in a counterfeit bill, surface bruises on Hass avocado fruits and transient changes in petri dish grown bacterial colonies. A fourth test used single band imaging in a set of standard laboratory analyzed plant samples, to quantitatively relate a red band light reflectance to its nitrogen content. These tests indicate that MEDUSA made images may yield qualitative and quantitative spectral information unseen to the naked eye, suggesting potential use in currency counterfeit tests, food quality analyses, microbial phenotyping and agricultural plant chemistry.

MEDUSA can be freely reproduced and customized from this research, making it a powerful and affordable analytical tool to analyze a wide range of subtle chemical properties in samples at industrial and science fields.

## Graphical abstract

**Figure:**
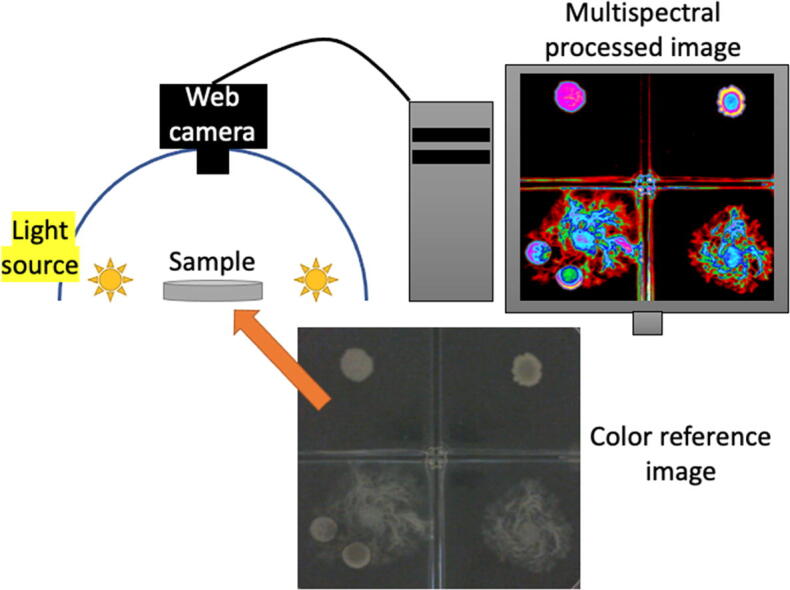

**Specifications Table**

**Table:** 

Hardware name	MEDUSA
Subject area	• Multispectral imaging • Open-Source hardware • Biological sciences • Environmental and agricultural sciences • Counterfeit detection • General surface analytical chemistry
Hardware type	• Imaging tools • Field measurements and sensors • Electrical engineering and computer science
Open-Source License	GNU General Public License (GPL)
Cost of Hardware	Less than 300USD
Source File Repository	https://osf.io/zhp4m/

## Hardware in context

Multispectral imaging is at the forefront of dry and non-invasive spatial analytical chemistry. It is used in fields as diverse as human health [9], food quality [8], plant science [22] soil chemistry [18], counterfeit [7] and art analyses [15]. Despite its proven general value, wide access to these systems is limited by complexity and a prohibitive cost linked to specialized imaging hardware and image analysis software.

A multispectral image shows a general 2d composition plot of an object. There, a sample of interest is illuminated with a wide spectral light source matching the spectral response of the camera sensor. To multispectral imaging a sample, its reflected light is split into multiple light bands which are used to produce as many images as bands are available. Depending on the physical and chemical making of a surface, each spectral image shows brighter or darker spots corresponding to lower or higher specific light band reflection spots. Quantitative composition on that surface can further be revealed by comparison to images of standard gold samples.

To better understand the making and use of these systems, they commonly comprise lighting and imaging modules, which produce specific light band associated images of an object. Each image is in turn made by pixels with variable intensity, whose tone is linked to spatial variations on the surface of that object. But capturing and analyzing so many pixels in spots and full images has a price, in which traditional hardware, software and regular statistics tools usually do not suffice.

Approaching this technology implies combining transdisciplinary skills and accessing expensive hardware not easily found in small enterprises or research settings, which limits its use. However, spectral images can also be made and analyzed by mixing narrowband LED sources, free image analysis software and common sensors found in consumer grade digital cameras. These common sensors may be considered as multispectral since they usually include filtering to capture red (R) green (G) and blue (B) light bands, by filtering the light before reaching the camera sensor. A mixed RGB image is then software made to create a human color illusion by presenting the digital image on an RGB screen.

Specific light band reflection results in “optical fingerprints” on an object image. While getting spectral information from RGB bands might be limited by a wide spectral band (full width at half maximum -FWHM- above 30 nm) and band superposition, cross RGB channel information from consumer grade cameras can perform like a standard grade spectroscopy system at analyzing medical samples [5].

LED made spectral imaging rely on specific band LED lighting instead light filtering. Such approach is already used by Videometer™, a commercial desktop multispectral system. Its specific use to nondestructively assess food quality and composition is found elsewhere [8]. It is a sophisticated device costing above 60.000USD whose usefulness to analyze surface chemical differences is widely demonstrated by literature records.

Quantitative image analysis software also eases multispectral imaging use. For instance, principal component analysis (PCA) reduces dimensionality of multiple images to make fewer images in which high variability spots are highlighted [12]. A pioneer paper on this topic unlocked the potential use of PCA for multispectral imaging to reveal hidden underdrawings and quantify pigment mixtures in antique art works [4]. PCA imaging analyses is freely available by using the plugin MSA514 running in the free software ImageJ [19].

We humbly consider that disclosing a customizable pipeline in which multispectral imaging automatically merges with PCA dimensionality reduction will ease the use of this technology and prompt discovery in many fields. However, deeper access to multispectral imaging requires not only a dropdown in hardware prices, but also simplifying its use and interpretation. Here we disclose the construction of a multispectral system, comprising electronic design, optical testing of components, and free software incorporation to automatically run a PCA dimension reduction on each set of spectral images without leaving the useŕs interface. Proof of concepts tests on microbiology, agriculture, food science and counterfeit analyses are also included.

## Hardware description

This multispectral camera (MSC), hereafter called MEDUSA, is a tabletop, customizable and cost-efficient multispectral imaging system built with less than a 300USD budget. It uses an open Python software framework and a hardware interfacing Arduino board. Building costs and user learning curve are a fraction of the required by any similar multispectral technology. The system also uses LED lighting from multiple VIS and NIR wavelengths spanning the visible and near infrared spectrum.

Quality of lighting and imaging components was tested by experimentation with research grade and custom-built standards. Two initial proof-of-concept tests included sets of spectral band images to analyze ink changes on a counterfeit bill, and surface bruises on Hass avocado fruits. These are followed by a scatter plot analysis of red band light reflectance as related to the nitrogen content of dry plant samples provided by a commercial plant analyses service. In addition beyond simple visual scoring on sample images, potential quantitative approaches are also illustrated by using principal component imaging dimension reduction on multispectral images of petri dish grown bacterial colonies. If properly calibrated, this device might find use for the non-invasive analyses on areas like:

• Food quality
• Counterfeit analysis
• Microbial colony phenotyping
• Plant chemical analysis
• Preliminary spectral analyses on multiple surfaces

**Fig. 1:**
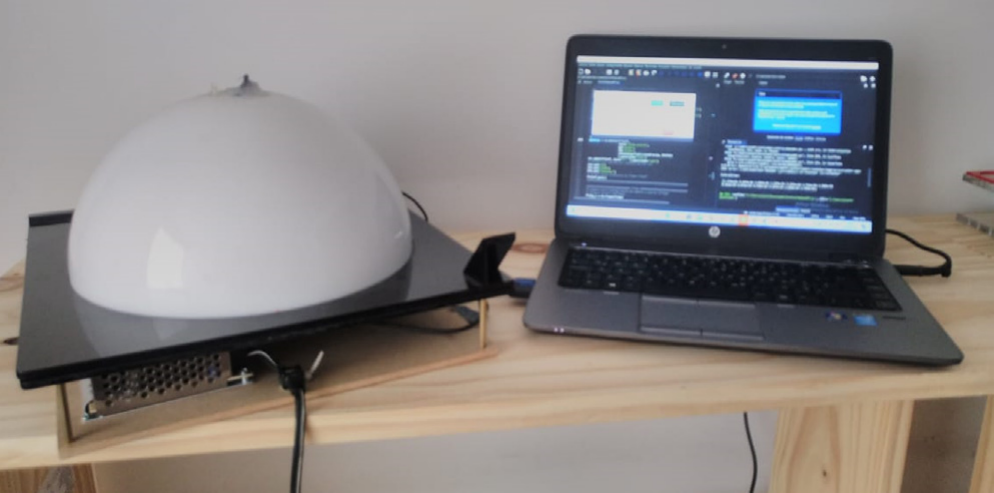
MEDUSA connected to a computer. At left, the upper semispherical dome and its opening frames are made from a Plexiglas sheet and are tied by a hinged joint. Lower space for electrical parts and circuitry is done by using four M3, 4 cm high standoffs between the lower semisphere frame and a wooden base.

The general appearance and main components of MEDUSA are shown in Fig. 1.

## Design files

### Design files summary

**Table:** 

Design file name	File type	Open-source license	Location of the file
UI_Medusa_PC_Python	Python file (.py)	GPL 3	https://osf.io/zhp4m/ - Python Script folder
ControlMedusaPC	Arduino file (.ino)	GPL 3	https://osf.io/zhp4m/ - Arduino Firmware folder
MEDUSA_AMEGA_Shield	Eagle file (.brd)	GPL 3	https://osf.io/zhp4m/ - PCBs folder
MEDUSA_Light	Eagle file (.brd)	GPL 3	https://osf.io/zhp4m/ - PCBs folder
MEDUSA_Frame P1 and P2	OpenSCAD file (.scad)	GPL 3	https://osf.io/zhp4m/ - Frame folder
MEDUSA_Baffle	STL file	GPL 3	https://osf.io/zhp4m/ - STLs folder

• UI_Medusa_PC_Python: Source code to the GUI operating MEDUSA.
• ControlMedusaPC: An Arduino firmware with routines to turn LEDs on and off from PC, this file is also used to modify the LED light intensity.
• MEDUSA_AMEGA_Shield: A custom Printed Circuit Board (PCB) which connects to the Arduino MEGA board and has additional connections for the lighting circuit.
• MEDUSA_Light: A custom PCB for soldering the needed LEDs.
• MEDUSA_Frame: Design for laser cut frames which supports the semisphere. It is made by two hinge joined frames allowing the user to open and close the imaging system.
• MEDUSA_Baffle: This part is a 3D print design for a baffle that blocks direct illumination of the camera sensor.

## Bill of materials

**Table:** 

Designator	Component	Number	Cost per unit – USD*	Total cost-currency	Source of materials	Material type
AMEGA	Arduino MEGA + USB cable	1	40	40	Amazon	Electronics
–	Power supply 12 V 3A (or similar)	1	12	12	Amazon	Electronics
–	Webcam HD3000	1	37	37	Amazon	Electronics
–	Plug and duplex wire (∼2 m)	1	2	2	General electronics store	Electronics
–	Ribbon wire (∼4m) + 12 JST male and female (each) connectors	1	4	4	General electronics store	Electronics
–	Ceramic capacitor (0.1 μF)	2	0.01	0.02	General electronics store	Electronics
–	Electrolytic capacitor (1 μF)	1	0.03	0.03	General electronics store	Electronics
–	Electric resistance (4.7 kOhms)	15	0.01	0.15	General electronics store	Electronics
–	Electric resistance (220 Ohms)	15	0.01	0.15	General electronics store	Electronics
–	2N3904 Transistor	15	0.02	0.3	General electronics store	Electronics
–	LM7805 Voltage regulator (or similar)	1	0.35	0.35	General electronics store	Electronics
–	40 pin male header	2	0.58	1,16	General electronics store	Electronics
–	40 mm M3 Female standoff + Screws	4			General electronics store	Electronics
–	2 pin terminal	1	0.58	0.58	General electronics store	Electronics
LEDS	Multiple band LEDs	45	0.55	24.75	General electronics store	Electronics
PCBSHD	MEDUSA AMEGA Shield	1	25	25	Local PCB manufacturer	Electronics
PCBLGH	MEDUSA AMEGA Light	3	10	30	Local PCB manufacturer	Electronics
FRP1	MEDUSA Frame P1	1	4.5	4.5	Local manufacturer	Plexiglass
FRP2	MEDUSA Frame P2	1	4.5	4.5	Local manufacturer	Plexiglass
–	Reflective paint “Pintuco™ – Blanco puro 1520”	1	5	5	Local hardware store	Vinyl based White paint
Baffle	Light direct blocking baffles	3	10	30	custom or local 3D printing service	PLA
			**Total**	**223.25**		

* Those prices are from year 2019 and may have changed over time.

AMEGA: Is a reference to the Arduino MEGA board.

PCBSHD and PCBLGH: The first has connectors with electronics and is installed onto the AMEGA; the second makes the LEDs pads.

FRP1 and FRP2: Both are part of a hinge mechanism that allows the user to open and close the semisphere.

Baffle: Is an accessory that may or may not be necessary depending on the user’s configuration and is used to block direct light to the camera. We provide a simple design, but the user can use a simple sheet of cardboard for this purpose. To avoid undesirable reflections on the sample area, it is white painted on its LED facing side and black painted on the sample facing side.

## Build instructions

Full assemblage of MEDUSA also requires 1) building the circuitry and the semisphere for even lightening and imaging 2) installing the software provided on the file repository and 3) programming the Arduino MEGA board with the firmware provided on the file repository.

### Building the lightening and imaging unit

**Fig. 2:**
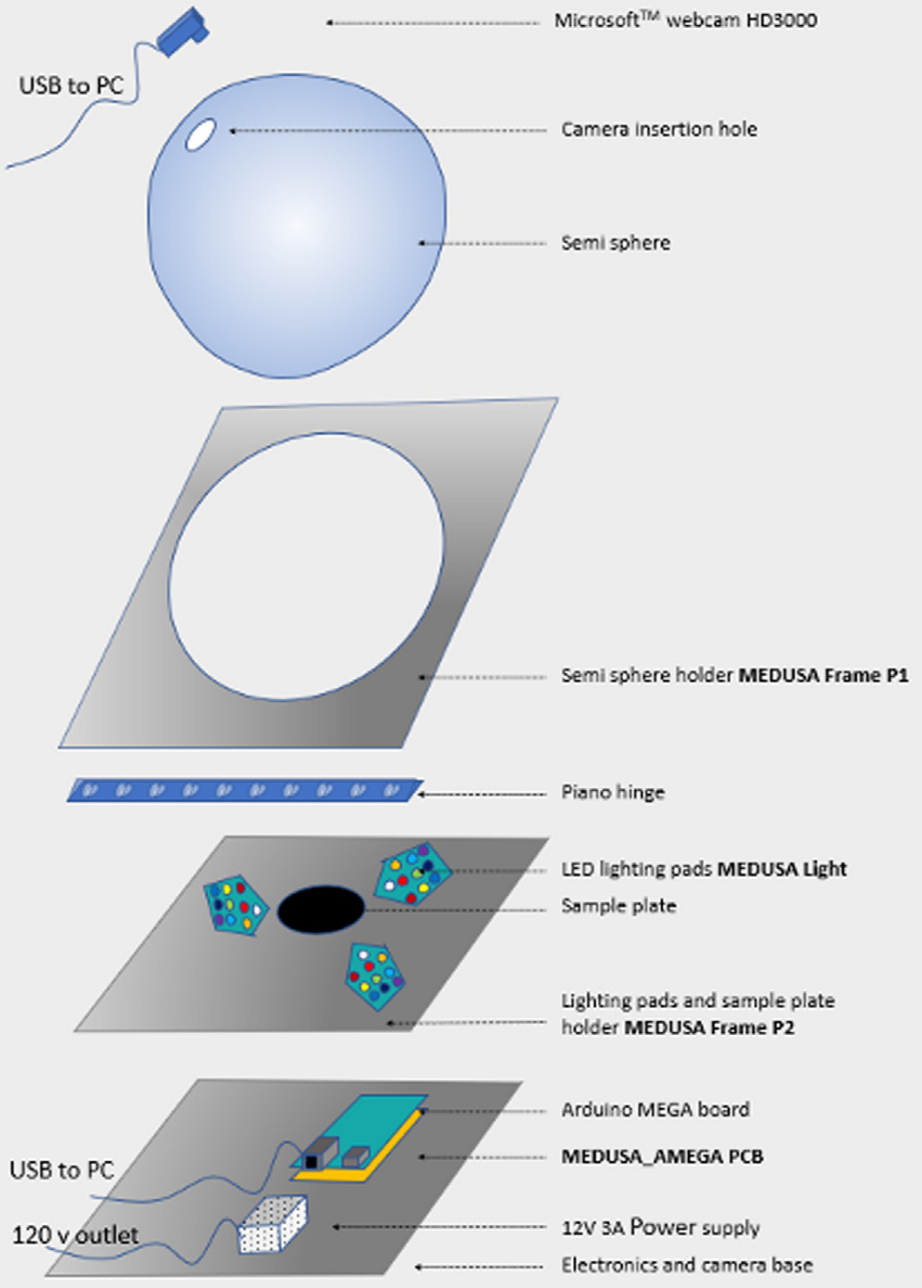
Medusa major components placement and external wiring connectors.

Each major component and placement is better visualized in Fig. 2. The system requires a semisphere, two PCBs (PCBSHD and PCBLGH) and electronical supplies available from online stores like Amazon. The documentation includes a detailed guide for assembling both circuits. The web camera is a videoconferencing HD3000 camera from Microsoft whose IR filter was removed to enhance its spectral sensitive range. The camera control is written in Python using the free library OpenCV [6], whereas the LEDs are controlled by the AMEGA shield.

### Building the electronics hardware

We posted a guide for assembling MEDUSA on the source file repository (OSF | MEDUSA: An ultra-low-cost webcam based multispectral imaging system) with the following topics:

1. Soldering all components onto PCBSHD and PCBLGH.
2. Installing PCBSHD on AMEGA and these to PCBLGH.
3. Putting MEDUSA together.
4. Adjusting light intensity.
5. Removing the camera IR filter.

We strongly recommend the reader to check out the assembly guide, especially the section about adjusting the LED light intensity. Here we use a PWM signal to modulate the LED intensity, as in many electronic devices that use this kind of signal to control the screen brightness. Adjusting this intensity can be needed for some specific band/sample combinations. For example, if a plant leaf sample is being analyzed you could need to increase the intensity of the blue or red associated bands which are strongly absorbed by chlorophyll.

The semisphere was built by hot molding a 2 mm thick Plexiglas sheet over a 14 cm radius CNC machined semisphere piece of solid wood. The inner reflective surface was prepared by first adding two layers of black paint which were further covered with three layers of white paint (Viniltex™ “Blanco puro 1520”).

This lighting device differs from an integrating sphere as presented elsewhere [10]. There, a light ray strikes the wall and lights up the entire inner surface after multiple scatterings. That device is bulky for our purposes and difficult to build. We instead used half of the integrating sphere, a semisphere, which was easier to build from any recipient with a spherical geometry and whose effectiveness for uniform lighting was tested before [11]. To improve light distribution in the region of photography (ROP), three light sources were placed at 8 cm from the center in a triangular pattern around the ROP. This pattern of light distribution was experimentally tested by comparing lightening variability inside a radius growing region of interest (ROI) on images taken with one, two or three LED sources.

**Fig. 3:**
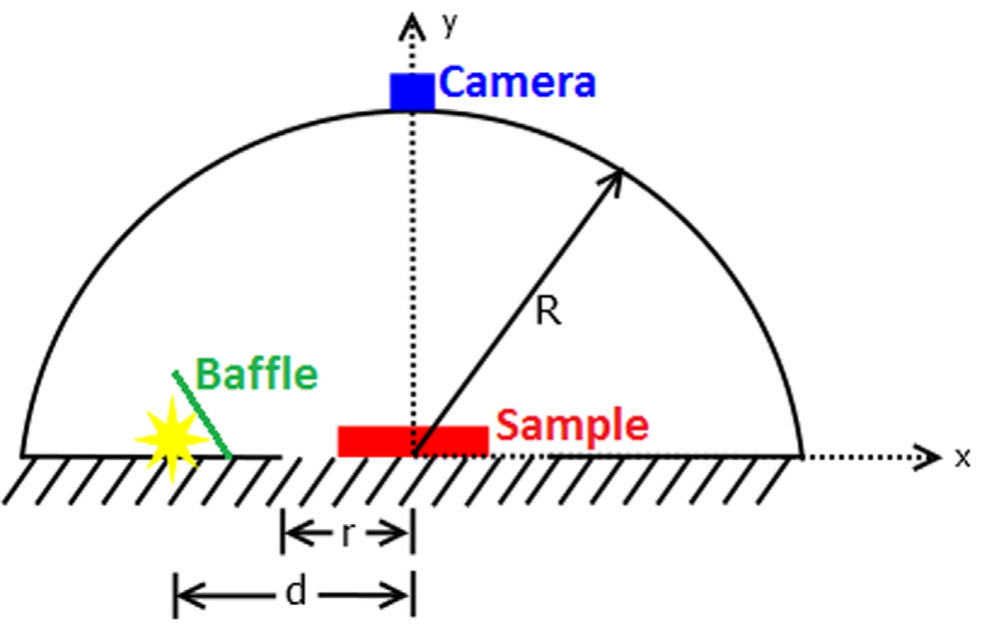
Optical configuration of MEDUSA with the relative position of its components. R, r and d are the radius of the semisphere, the radius of the region of photography and the distance of the light source (represented by a star) to the center respectively; 14, 6 and 8 cm in our settings. The baffle blocks direct light from reaching the camera sensor.

Fig. 3 presents a lateral view of the semisphere and the relative positions of components. The camera was directly inserted above the ROP as seen in Fig. 3. The orientation of the camera should be tested by the user right before fixing it to the semisphere by previewing an image.

The baffle shown in Fig. 3 could be different, the design provided on the file repository called MEDUSA_Baffle is just something we propose as a possible solution for someone getting direct light to the camera sensor. Different geometries or different solutions must be defined by the user.

### Installing the software and camera configuration

MEDUSA G is the camera and LEDs easy control software. Python is initially installed through Anaconda [1]. Then additional libraries must be installed; Pyserial [13] which communicates the user interface with the AMEGA board. OpenCV [6] which communicates the user interface with the camera. The camera is used on auto mode, except that python scripting is used to convert RGB images to gray scale images. Additional settings can be customized by skilled python users. The AMEGA board must be programmed with the firmware Medusa_PC_Arduino. The final step is to run the script UI_Medusa_PC_Python within the environment of Anaconda, which is bundled and runs along with Spyder [21].

## Operation instructions

### Connection to PC and powering MEDUSA

The connection only requires an AC plug which is connected to a 12 V supply voltage and two USB cables that are plugged to the PC, one for the camera and the other for the AMEGA board.

### Using the software

**Fig. 4:**
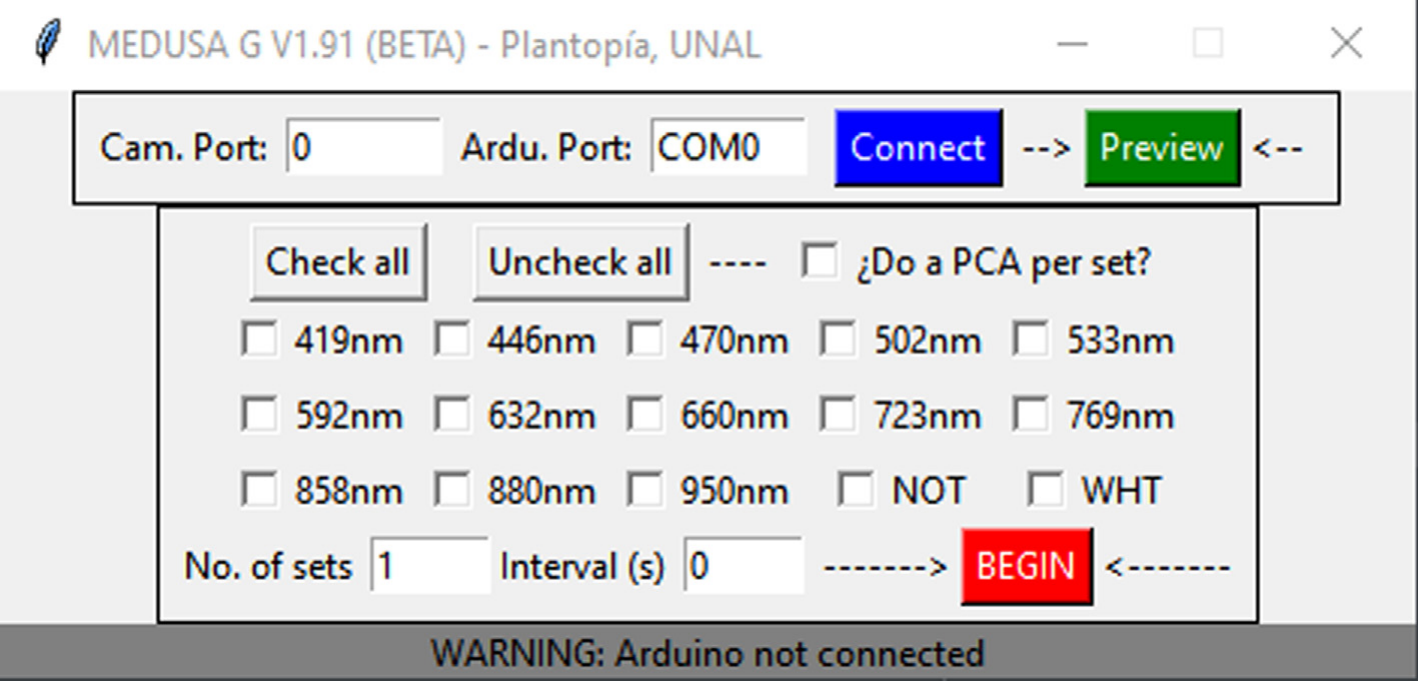
Appearance of the MEDUSA G User Interface written in Python to control MEDUSA.

MEDUSA G is presented in Fig. 4, it was written in Python using the graphic library Tkinter [20], which is included by default with any Python distribution. Additionally, it depends on the free libraries pyserial [13] to communicate with the AMEGA board, and OpenCV [6] to communicate with the camera.

MEDUSA G was developed under a Windows environment, but since it was written in Python It can be user modified to run under a different operating system. Its functions can be divided into 5 groups:

1. **Cam. Port** and **Ardu. Port** are connecting port numbers for the camera and AMEGA board. These are integers ≥ 0. For instance, at the Arduino code COMX, X is an integer ≥ 0. **Connect** links the camera and the AMEGA to the PC. **Preview** pops up a preview a default white led illuminated image. Note that these functions will work only if the camera and AMEGA board are connected to the PC.
2. **Check all/Uncheck all** Buttons allows mass selection of bands instead of manual band checking. **Do a PCA per set** will make a dimension reduced PCA image recovering most variation across each set of images and pixels therein.
3. **NOT and WHT** checkboxes within the light bands are just positions with no LED installed or a white LED lighting to make a reference VIS image.
4. The “Do a PCA per set?” checkbox will perform a Principal Component Analysis (PCA) over each set of captured images.
5. **No. of sets** and **Interval (s)** are used together to run planned time lapse experiments. Sets of images in selected bands, separated by a time interval specified in seconds are user defined. Finally, the **BEGIN** button starts the experiment and is turned into a STOP button once clicked.

The images captured, as well as the dimensionally reduced PCA images highlighting the image set variance associated pixels, are automatically stored in the same folder in which the software file is located. Note that all images are converted to gray scale .png files except the color which is a traditional RGB .png file. The gray conversion comes by default on the OpenCV library by using the function BGR2GRAY. Additionally, advance python knowledge may allow the user to configure some camera parameters such as white balance, exposure, etc. We used the camera in automatic mode for all our tests. Except for color reference images, any other file is changed to a gray scale image.

## Lighting and imaging performance

Below we describe the process to characterize these components in MEDUSA and its validation through some laboratory experiments.

### Quality of LED lighting

**Fig. 5:**
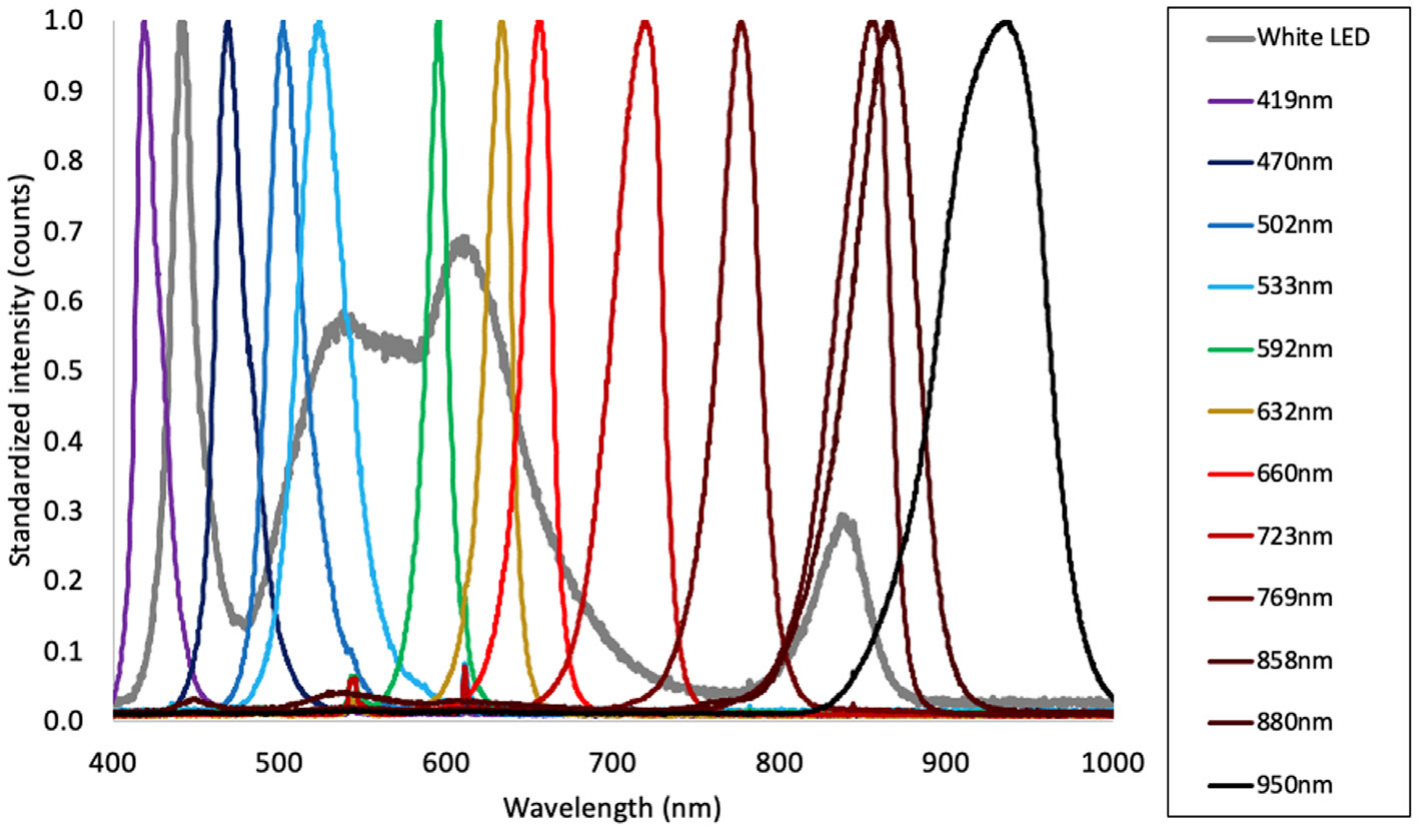
Standardized reflectance spectra of available LEDs. Spectra were obtained by reading the reflection of LED light upon a certified reflectance standard from Labsphere™. Such readings were captured using an OceanOptics™ FLAME. LED sources are named by the wavelength value at its peak’s spectrum.

Overall LED bands spectra covered visible (VIS) and near Infrared (NIR) electromagnetic spectrum as seen in Fig. 5. These bands have on average a FWHM ± 10 nm and were captured in a dark room by reflection on a Labsphere™ certified reflectance standard (CRS) using a FLAME-VIS spectrometer and a halogen light source (both from OceanOptics™).

### Testing the semisphere and the inner light reflecting surface

**Fig. 6:**
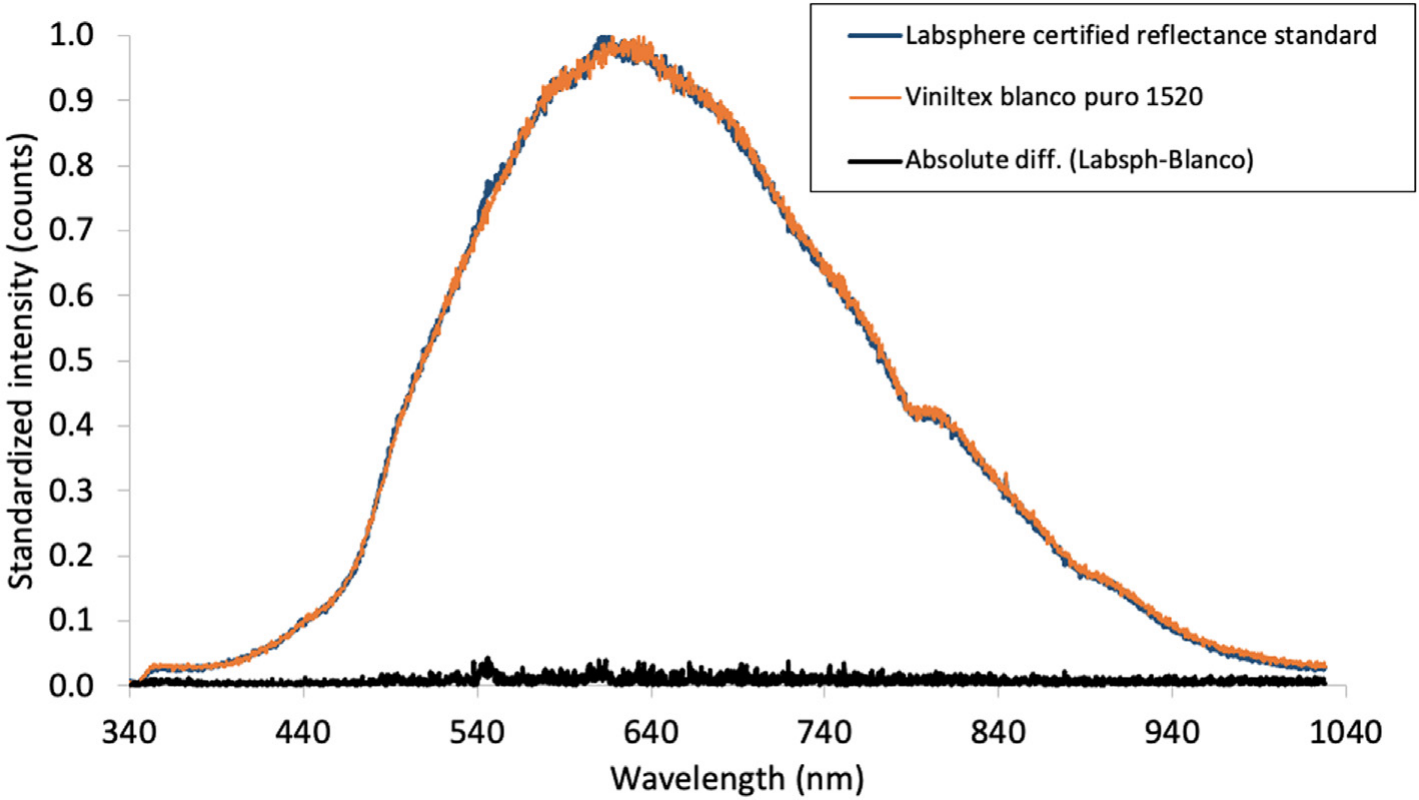
Standardized spectra of a halogen lamp (HL-2000 FHSA, OceanOptics™) reflected from a certified reflectance standard (Labsphere™) and from the white paint (“Blanco puro 1520”, Viniltex™). The reflected light spectra were captured with a FLAME-VIS spectrometer from OceanOptics™. The black line spectrum shows the absolute difference between the standard and the white paint reflectance.

The reflectance spectrum from a halogen lamp (HL-2000-FHSA, OceanOptics™) on “Blanco puro 1520 Viniltex™” white paint matched well the spectrum reflected by a Labsphere™ CRS. Fig. 6 presents the superposed reflected spectra which were almost identical. To better visualize the differences, an absolute difference plot between the standard and the white paint reflectance is also included.

### Testing the uniform lighting in the sample area and the spectral band sensitivity of the USB camera

Four parameters to guarantee image consistency were identified, likewise, three tests were designed to test some of these parameters.

1. Absence of light in dark conditions (or background noise): To remove this source of error, the camera was programmed to capture a dark, LEDs off image, which is then subtracted from its subsequent LED illuminated image.
2. Even light distribution: It guarantees spatial consistency of the lighting system. Here, a pink paper 6 cm in diameter was positioned in the center of the ROP. Three tests were run after lighting with 1, 2 or 3 632 nm centered LEDs respectively. The images were converted to a gray scale and then false colored with a 6 Shades color palette in ImageJ to visualize the differences. These differences were in turn quantified by calculating the mean gray value (MGV) and standard deviation (SD) of pixels in a 0.1 mm successive radius growing circle, starting from the ROI center up to a 70 mm final radius, such measurements were made with simple ImageJ scripting.
3. Sensor spectral band sensitivity: The camera sensor should be sensitive to the available LED bands. To test this, the CRS from Labsphere™ was placed in the center of the ROP and a set of images was captured using the available LED bands. Then, the MGV from each image was obtained from a circular ROI inside the CRS. Sensor saturating bands evident in images with MGV ≥ 255 were intensity reduced to reach MGV values lower than 240. As a comparison, the intensity perceived by a camera, side placed optical fiber FLAME-VIS spectrometer from OceanOptics™ was also measured.
4. Consistent lighting and imaging performance over time: Time lapsed images of a custom standard, which are captured under the same lighting conditions, must show the same reflectance. To test this, a pink paper disk 6 cm in diameter was placed in the center of the ROP. Then 100 images were sequentially captured at 10 s intervals using a 525 nm band centered LED. From each image, a circular ROI was located at the exact same position to get their MGV.

#### Even light distribution

**Fig. 7:**
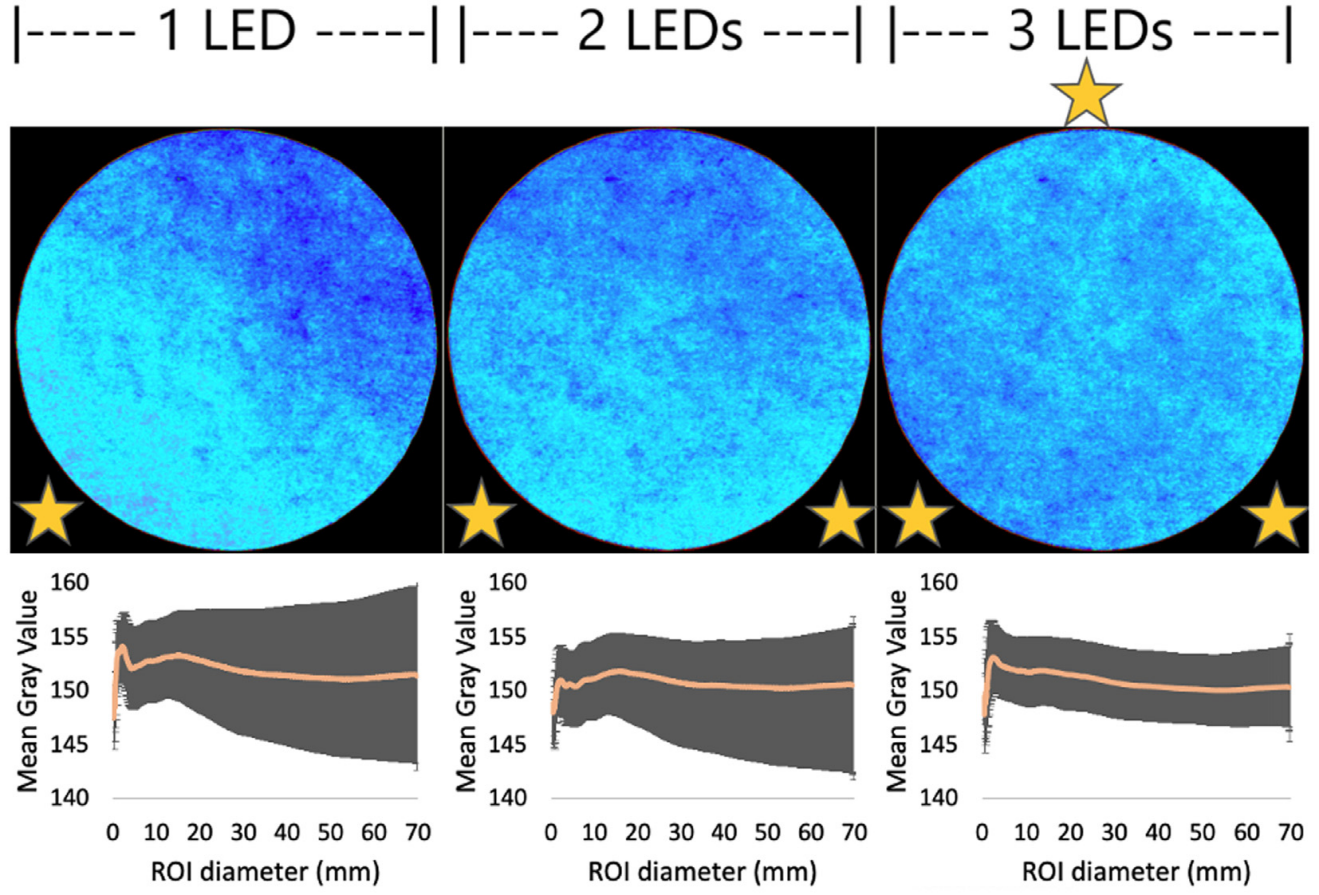
Light intensity over a 7 cm disc of pink paper as light sources are increased from 1 to 3 LEDs. For each case, the light intensity profiles were obtained by measuring the MGV (represented by the orange line) inside a growing circular ROI, the shadowing region represents the corresponding SD for each MGV measurement. The relative position of LEDs is represented by a yellow star. (For interpretation of the references to color in this figure legend, the reader is referred to the web version of this article.)

Fig. 7 visualizes** the light distribution over a 7 cm in diameter pink paper disk as the number of lightning LEDs is increased from 1 to 3. The images were taken using a 632 nm band centered LED. Additionally, a light distribution profile was obtained by measuring MGV values along with their SD inside a growing circular ROI centered with the paper.

Light distribution was improved as the number of LEDs is increased from 1 to 3. Subtle lighting differences are image visualized using a *6 Shades* color pallet available on ImageJ. It is also reinforced by the MGVs and SD represented by the distribution profile plots below each image. Since the LEDs are plugged in a parallel circuit configuration their light intensity (measured as MGV) does not change as the number of sources is increased. The decrease on the SD values is then linked to an increased number of LEDs. Even though it does not appear to follow a proportional relationship, a 3-LED configuration distributed in a triangular pattern was chosen here, as the variations seems to become constant.

It is worth noting that further improvements to asses general lightening design by diffusive domes are already available [11] and could be custom added to MEDUSA or its images by interested users. Geometric and optical adjustments are also user customizable to obtain multispectral transmittance and fluorescence imaging of diverse objects. Although all possible configurations and improvements are beyond our purpose of deploying the basics of an open and customizable multispectral imaging and analysis platform.

#### Sensor spectral band sensitivity

**Fig. 8:**
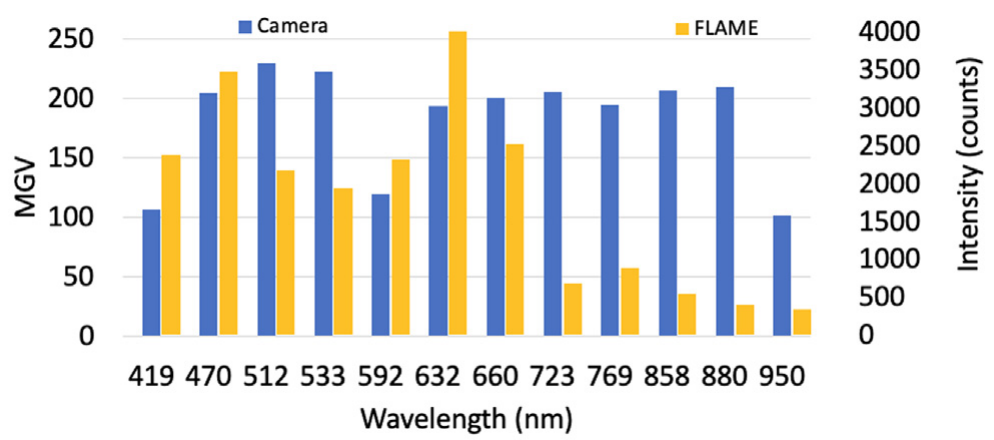
LED band reflectance intensity as perceived by the Webcam HD3000 from Microsoft™ with infrared filter removed and a FLAME VIS-NIR spectrometer from OceanOptics™. These mean gray values were taken by placing a Labsphere™ certified reflectance standard on the measuring area.

The camera sensor must be sensitive to the available LED bands. A set of images was captured targeting the CRS from Labsphere™. Since some LEDs saturated the camera sensor, their reflectance intensity was reduced to a maximum of 240 MGV to avoid data loss, this process is detailed on the assembly guide. Fig. 8 presents a reference reflectance “spectrum” for MEDUSA, which might be considered in case this system is replicated. For comparison, the intensity perceived by a FLAME-VIS spectrometer from OceanOptics™ was also captured. It clearly shows a relative high sensor response on most bands, although it is lower for bands at 419, 592 and 950 nm. Experimentation and deep literature review will be required to define the spectral bands to use according to specific targets. Nonetheless, the lighting pads are freely customizable. Please consider also that the camera resolution is 8 bits while the FLAME’s resolution is 16bits, hence, the scale difference is not proportional to their specific sensitivity.

#### Consistent lighting and imaging performance over time

**Fig. 9:**
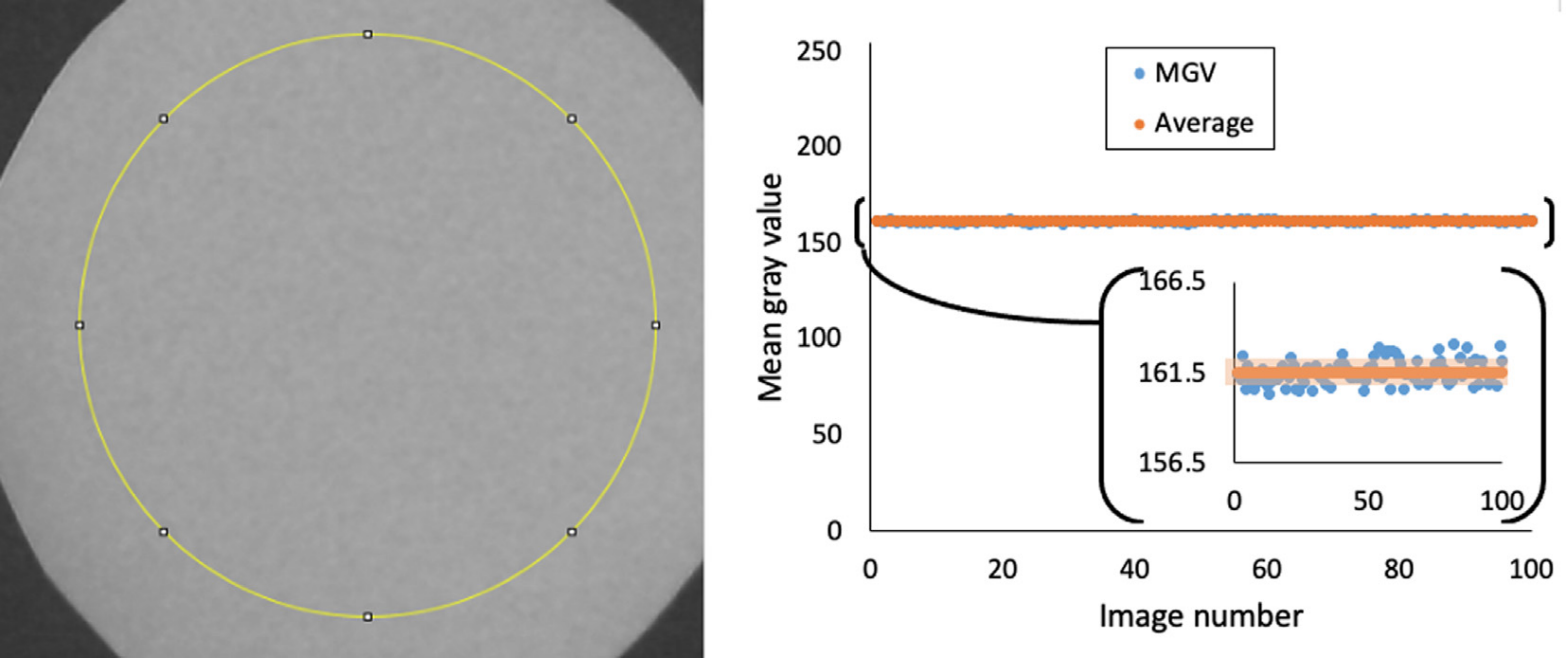
Consistency test where the mean gray value was measured in 100 sequential images under the same lighting conditions. The LED band was centered at 525 nm and a pink paper 7 cm in diameter was used as a target. The yellow circle shows the region of interest for this test measurements. (For interpretation of the references to color in this figure legend, the reader is referred to the web version of this article.)

The webcam HD3000 made a set of 100 consistent images over time. These images were automatically captured at a 10 s interval and converted to grayscale. Fig. 9 shows a set SD lower than 0.5% on the MGV of a 7 cm radius circle. It shows that this camera can consistently capture sequential images at least for short term experiments. Additional experimentation over longer periods of time might be needed for specific users.

## Proof of concept tests

We did not make comparative tests against commercial desktop multispectral imaging systems since these were not available. The overall performance of MEDUSA beyond its lightening and imaging components were experimentally tested through four proof-of-concept tests. Test one is made with the total number of bands taken from a fake and authentic bill, as well as the regular RGB image. Test two is comprised by a multi band image montage of varying quality avocado fruits; it compares the regular RGB image with several IR imaging bands. Test three shows the potential analytical value of single band imaging. There, a 660 nm band was used in a set of standard laboratory analyzed plant samples, to quantitatively relate a red band light reflectance to its nitrogen content. Finally, a fourth test used multiple imaging bands dimensionally reduced by multivariate statistical analysis. PCA images were obtained by python scripting which is provided here, and are presented as dimensionally reduced 1st PCA (PCA1) image components. Final pseudo color images were obtained in the free software ImageJ [19].

### Detection of counterfeit bills

**Fig. 10:**
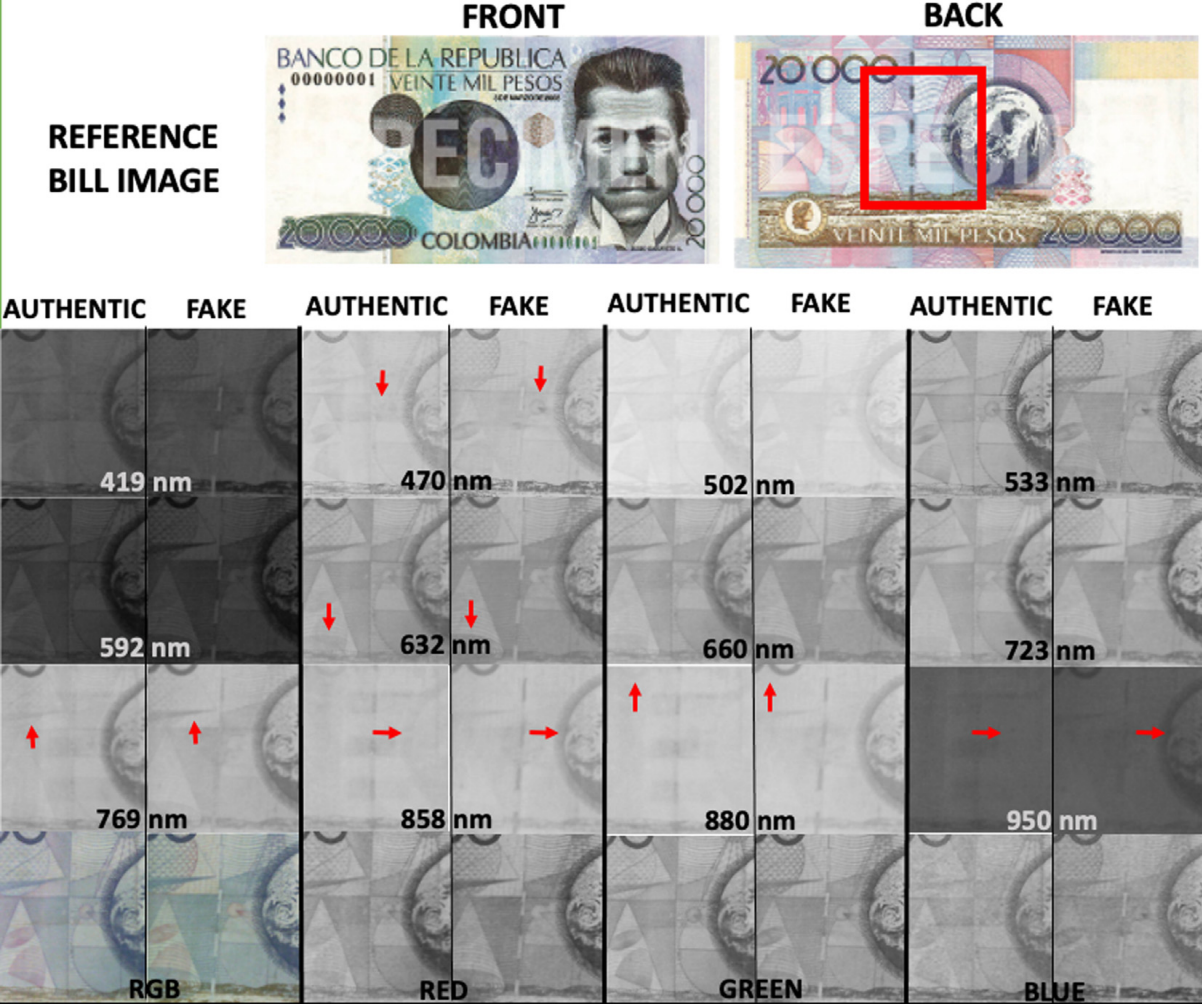
Montage of fake and authentic 20.000COP bills placed on the same positions and at the same distance from the camera. The red square highlights the region of interest. Red arrows within each imaging band indicate dissimilar ink areas in the authentic and the fake bill, only a few point differences are highlighted. (For interpretation of the references to color in this figure legend, the reader is referred to the web version of this article.)

MEDUSA was used to detect general surface ink differences. Fake and authentic 20.000COP bills were multispectral imaged and a comparative montage was made using the available bands and the color image along with its corresponding red, green and blue channels (Fig. 10). Some of the bands (e.g., 880 nm or 950 nm) clearly show differences between the authentic and fake bill. These are not easily noticeable on the RGB image.

### Avocado fruit damage detection

**Fig. 11:**
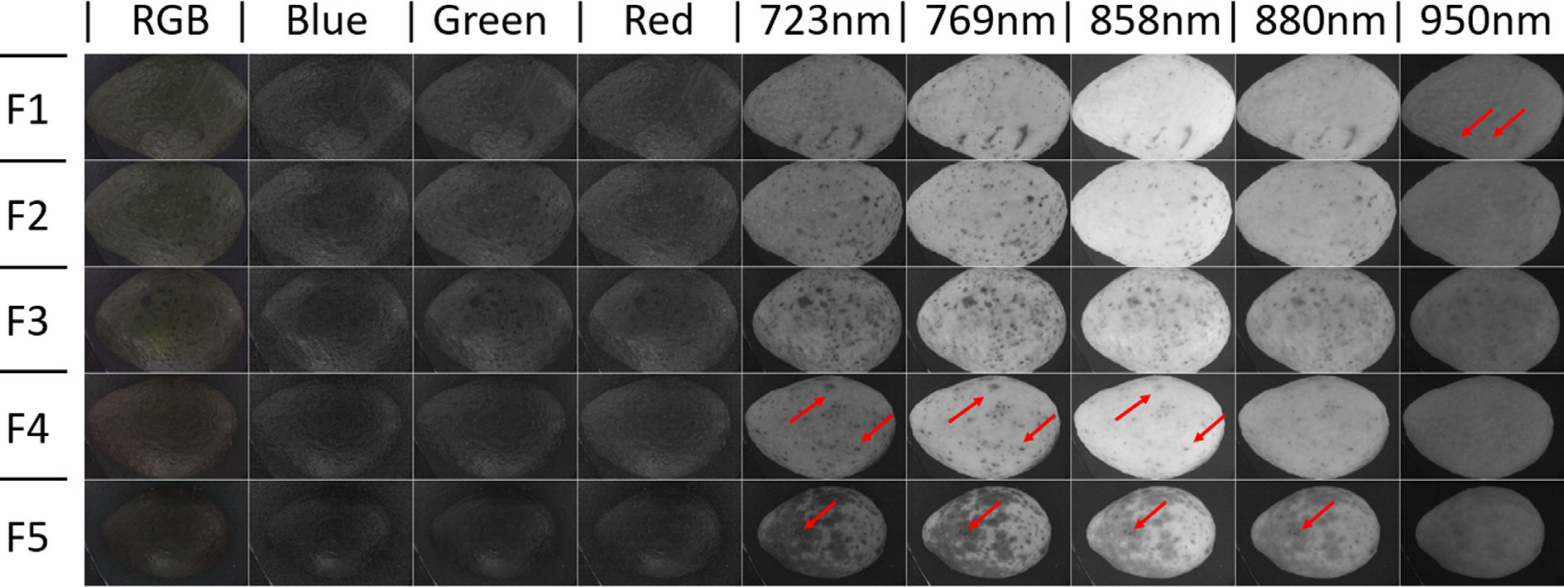
Montage of five quality and ripening increasing (from bottom to top) hass avocado fruits placed on the same position and at the same distance from the camera. Red arrows highlight specific band damages unseen on the standard RGB color and spare RGB bands. Previous advantages on fruit quality detection by multispectral imaging on thick rind fruits like avocados were presented before [2]. (For interpretation of the references to color in this figure legend, the reader is referred to the web version of this article.)

Five avocado fruits (*Persea americana cv.* Hass) with different amount of damages and increased ripening stage (bottom to top) were obtained from the local market and externally imaged on MEDUSA. A montage was made using the RGB and IR bands. The latest highlighted different surface damages (Fig. 11). Some red arrows are used to pinpoint damages better seen on specific bands.

Fig. 11 suggests that a color image provides few clues on general fruit quality. However, specific band images show remarkable fruit differences at their skin. Some of these differences might be explained by differences in fruit ripening. Previous research has also shown chances for non-contact estimation of avocado ripeness at harvest, as reported by [23], which made a comprehensive study on avocado ripening detection by using a scientific grade hyperspectral camera, industrial lighting and refined image analysis algorithms in an external software platform.

### Red light reflectance and nitrogen content in leaf plant samples

**Fig. 12:**
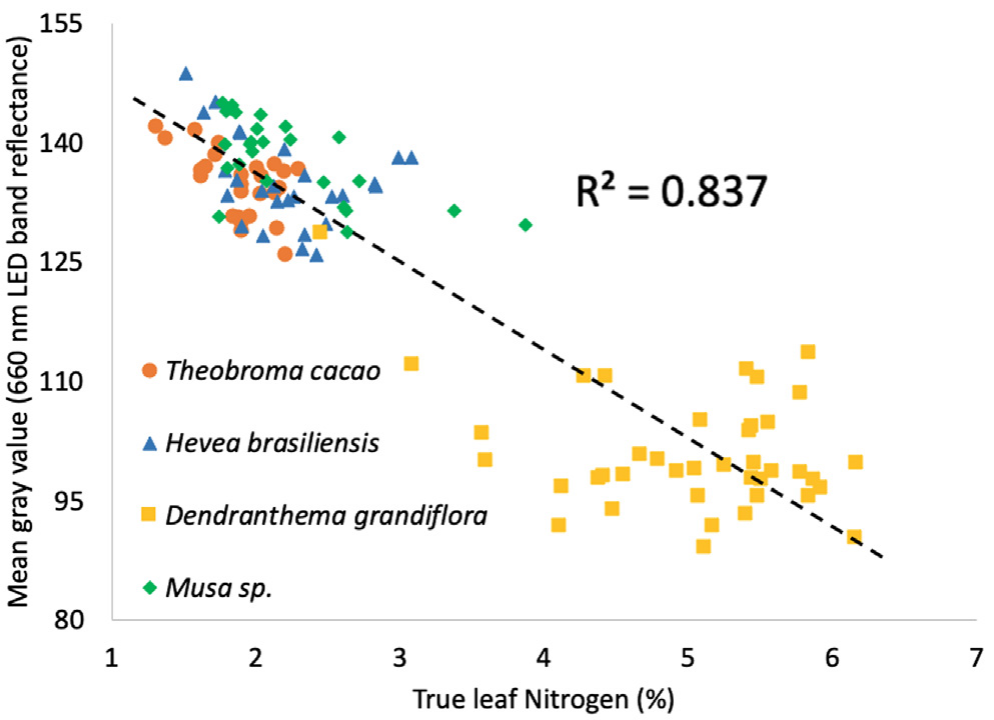
Medusa obtained 660 nm light band reflectance on dry ground plant samples, and its grossly association with its Kjeldahl extracted nitrogen content. Mean gray values were extracted in ImageJ using a macro function freely available elsewhere [16].

Nitrogen in plant leaves can be estimated from a 650 nm leaf light transmittance standard meter known as a SPAD meter [25]. Transmittance is tightly tied to surface reflectance. To preliminary check the possibility to estimate nitrogen concentration in dry ground plant samples by red light reflectance, we made 120 reflectance images using a 660 nm centered LED band in MEDUSA. These samples comprised leaves from four commodity crops (cocoa, natural rubber, chrysanthemums and bananas) which were provided by a commercial plant analysis laboratory and were previously analyzed by a standard Kjeldahl nitrogen test. It showed a strong negative correlation between increasing leaf nitrogen content and dry ground sample imaging decrease in red light reflectance (see Fig. 12). It was expected from well settled theory on optical spectroscopy for plant nutritional analysis, in which increased red light reflectance associates with a decreased leaf nitrogen content [17]. This suggests a potential for MEDUSA to dry analyze nitrogen and possibly other plant nutrients if further statistical models are properly developed and validated.

### Bacterial colony analysis

**Fig. 13:**
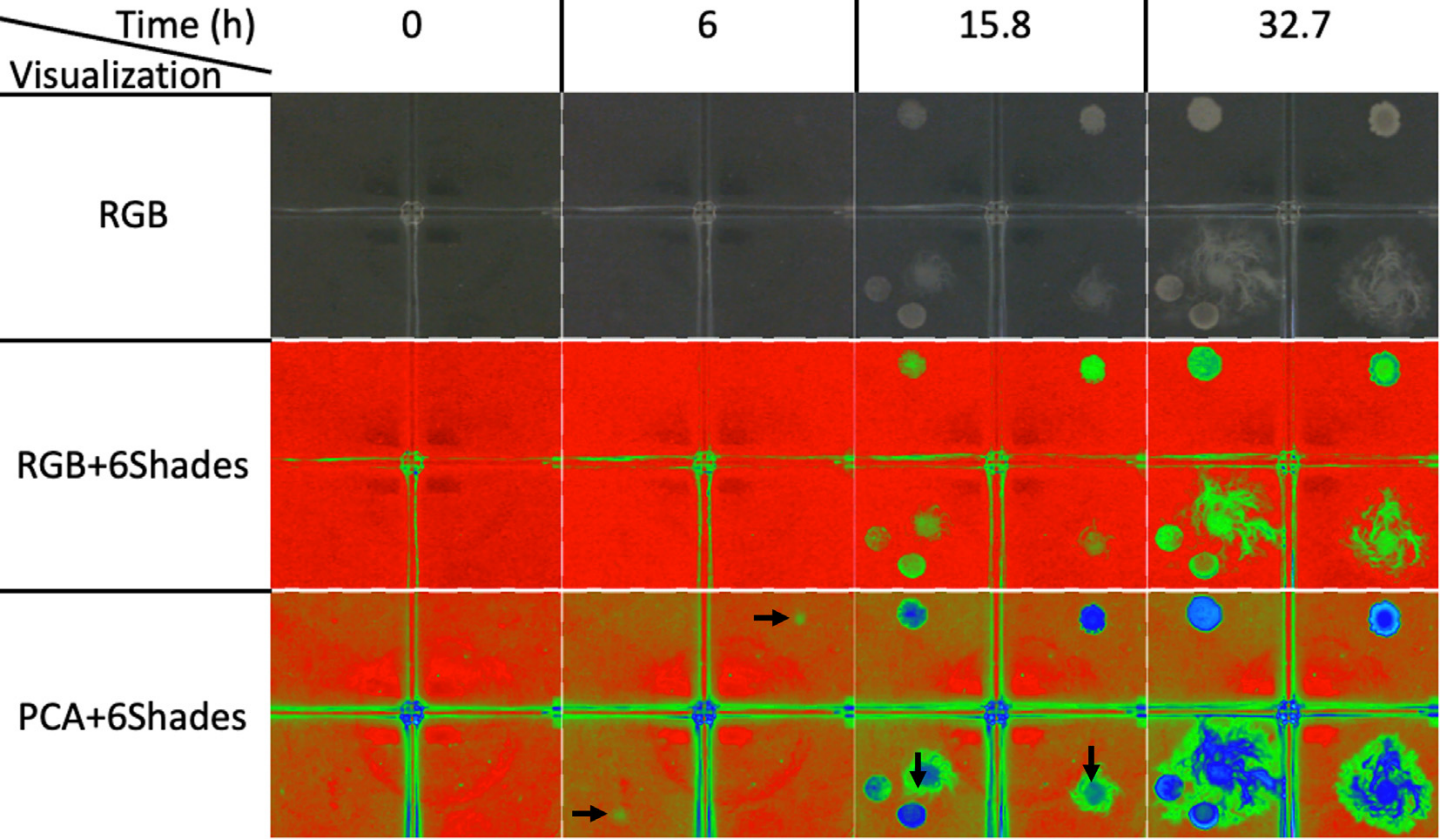
Time lapse analysis of bacterial colonies over 4 development stages and 3 types of visualization. Freshly seeded (t = 0 h); Colonies not yet visible with RGB (t = 6 h); Visible on both RGB and PCA1 images (t = 15.8 h); End of test with interacting colonies (t = 32.7 h). Horizontal arrows track specific time colony changes undetected by a traditional RGB image. Vertical arrows show differences in inter colony changes as perceived from RGB and PCA images.

Tools for automatic bacteria colony growth analysis might help to understand strategic shifts in microbial metabolism with clear biological [14] and human health consequences [3]. These shifts are also linked to chemical clues or media changes which can be detected by sophisticated, yet invasive techniques [24]. Here, we multispectral imaged bacterial colonies seeded on a 4-compartments Petri dish (see Fig. 13). Such colonies were originally obtained from a sterile water dilution of a garden soil sample. The test was conducted on 5 mL of nutrient agar on each dish section.

Inoculum was a 5 μL drop of a bacterial cell suspension which was stored in a 0.85% sterile NaCl solution. The Petri dish was placed open inside the multispectral chamber at room temperature. The test was carried out in the Soil Microbiology Laboratory of the National University of Colombia at its Medellín campus, which has an average temperature of 22 °C and a relative air humidity ranging between 63% and 73%. A set of images was then obtained at 10 min interval during 33 h. We chose 4 specific times to highlight the differences between the RGB images and the PCA multispectral images (Fig. 13).

Colonies on the upper left and right compartment were UV fluorescent on King B agar, made by gram negative bacilli, presumably identified as *Pseudomonas* sp. The diffuse grown colony on the lower right compartment is made by gram positive, spore forming bacilli, and is presumably identified as *Bacillus* sp. These colonies are used only to test the system. We expect other microorganisms to exhibit a variable development which can be in turn traced by this multispectral imaging. Some areas in which differences are detectable over time are pointed with vertical black arrows and some areas where changes were only visible with the PCA1 image are pointed with horizontal black arrows.

## Device limitations

Here we disclose a fully open spectral imaging system, which can be adjusted for specific purposes and sample types. Not without drawbacks, our experimental tests show that the sensing and illumination system, based on regular LEDs and a common web camera, can perform well in lapsed runs of sample imaging. As for any analytical tool, MEDUSA must be calibrated by the user. Then it might be necessary to make reflectance standard photographs with all light bands to assure operating consistency and check for suspicious reflection values before using the system. To ease that task, instructions on LED intensity adjustment are also included in the assembly guide.

It is worth noting that in spectroscopy applications in general, adequate specific band light intensity depends on both: the sample optical properties being analyzed as well as the sensor sensitivity in these light bands. Biases will be avoided by keeping a consistent optical set up. Then we recommend the user to do some previous experimentation before defining the more convenient band intensity to use. The proof of concept tests presented here helped our point on showing the potential discovery of this open tool, but these are only glimpses which must be increased in sample numbers before drawing any conclusion. PCA images yielded easy examples for visual scrutiny, which may inspire the design of further standard analysis systems for samples of interest to builders and users of this system.

For instance, it is apparent from the correlation between light absorption and N concentration measured in the laboratory, that further increasing on plant analysis in this system may contribute to building new tools for plant nitrogen dry analysis, based on a few or even a single light band. These however are ambitious goals which are just beyond the scope of this system design and preliminary test

Specific enhancements can be directed to:

• Adding more bands with a narrower FWHM.
• Using a monochromatic camera with increased spectral sensitivity and speed.
• Custom software programming and processing guided by hypothesis might also provide new insights into many nature’s hidden clues.

Further use may also reveal additional downsides requiring attention. A remaining challenge for a system like MEDUSA is the speed at which some essential components can change on the market. For instance, there is a big chance of not finding the very same LEDs used in MEDUSA if we were to look for them tomorrow or the day after. Although the same is true in many DIY projects. Then PCA images might not be exactly reproduced in a different set up of this system, since changes in LED intensity and spectrum, general hardware differences and even environmental conditions might translate into a different image of a same object. However, dry and contactless variations on samples surface will be easily discovered by specific interactions between light energy and objects of interest if this multispectral tool is available, and their components properly tested.

True access to tools fuels advances in science and technology. Despite its downsides, MEDUSA is an open and affordable alternative to speed discovery and can be build and used in many fields by small, transdisciplinary teams, without the hassles of the rigid and cost prohibitive access to commercial devices.

## Declaration of Competing Interest

The authors declare that they have no known competing financial interests or personal relationships that could have appeared to influence the work reported in this paper.
